# Src-Like Adaptor Protein (SLAP) Binds to the Receptor Tyrosine Kinase Flt3 and Modulates Receptor Stability and Downstream Signaling

**DOI:** 10.1371/journal.pone.0053509

**Published:** 2012-12-31

**Authors:** Julhash U. Kazi, Lars Rönnstrand

**Affiliations:** Experimental Clinical Chemistry, Wallenberg Laboratory, Department of Laboratory Medicine, Lund University, Skåne University Hospital, Malmö, Sweden; University of Iowa, United States of America

## Abstract

Fms-like tyrosine kinase 3 (Flt3) is an important growth factor receptor in hematopoiesis. Gain-of-function mutations of the receptor contribute to the transformation of acute myeloid leukemia (AML). Src-like adaptor protein (SLAP) is an interaction partner of the E3 ubiquitin ligase Cbl that can regulate receptor tyrosine kinases-mediated signal transduction. In this study, we analyzed the role of SLAP in signal transduction downstream of the type III receptor tyrosine kinase Flt3. The results show that upon ligand stimulation SLAP stably associates with Flt3 through multiple phosphotyrosine residues in Flt3. SLAP constitutively interacts with oncogenic Flt3-ITD and co-localizes with Flt3 near the cell membrane. This association initiates Cbl-dependent receptor ubiquitination and degradation. Depletion of SLAP expression by shRNA in Flt3-transfected Ba/F3 cells resulted in a weaker activation of FL-induced PI3K-Akt and MAPK signaling. Meta-analysis of microarray data from patient samples suggests that SLAP mRNA is differentially expressed in different cancers and its expression was significantly increased in patients carrying the Flt3-ITD mutation. Thus, our data suggest a novel role of SLAP in different cancers and in modulation of receptor tyrosine kinase signaling apart from its conventional role in regulation of receptor stability.

## Introduction

The Fms-like tyrosine kinase 3 (Flt3) is a type III receptor tyrosine kinase (RTK) belonging to the same subfamily as the platelet-derived growth factor (PDGF) receptors, cKit, and the macrophage colony stimulating factor (M-CSF) receptor [1]. Wild-type Flt3 is primarily expressed in hematopoietic progenitor cells and functions in hematopoiesis. It normally promotes proliferation and differentiation of hematopoietic stem cells and progenitor cells through the activation of mitogen-activated protein kinase (MAPK) as well as phosphoinositide 3 kinase (PI3K)-Akt signaling pathways. Stimulation of Flt3 receptor with its ligand (FL) leads to dimerization of receptors and activation of its intrinsic tyrosine kinase activity. This event further initiates phosphorylation on tyrosine residues within the receptor intracellular domain, as well as on downstream signal transduction molecules. The phosphorylated tyrosine residues facilitate high affinity binding of signal transduction molecules containing Src homology 2 (SH2) or phosphotyrosine binding (PTB) domains.

Src-like adaptor protein (SLAP) was initially identified as a negative regulator of growth factor signaling [2] and subsequently implicated in negative regulation of T-cell signaling [3]. SLAP is highly homologous with Src family kinases (SFKs) containing Src homology 3 (SH3) and SH2 domains. Unlike Src, SLAP lacks a tyrosine kinase domain and, instead, contains a unique carboxy terminus. T-cell signaling is regulated by association of SLAP with the phosphorylated T-cell receptor (TCR) leading to recruitment of the E3 ubiquitin ligase Cbl to the TCR. This lead to ubiquitination and degradation of the TCR [4]. Cbl is one of the most studied ubiquitin ligases involved in RTK signaling. Loss of function mutations in Cbl have been described in acute myeloid leukemia (AML) and been shown tocontribute oncogenic transformation [5]. Although originally implicated as a regulator of T-cells signaling, SLAP is also involved in regulation of hematopoietic cell signaling. SLAP also associates with components of the B-cell receptor (BCR) complex and modulates downstream signaling by Cbl-mediated down regulation of the complex [6].

Approximately 30% of patients with AML carry a mutation in the Flt3 gene [7]. Mutations result in ligand-independent constitutive activation of the receptor. The most common mutation is the so-called internal tandem duplication (ITD) in the juxtamembrane region of the receptor [8]. The ITD mutation disturbs the function that the juxtamembrane region of receptor poses on the kinase domain. Other mutations that can induced constitutive kinase activity include point mutations in the vicinity of codon 835 or 842 within the tyrosine kinase domain have been described in both pediatric and adult AML [9].

Signals from Flt3 aretransmitted through direct association of signaling molecules to the activated receptor. Relay molecules reported to be recruited include SFKs, SHIP1, SHP2, Cbl, Ras-GAP, PLCγ, Vav, Shc, Grb2 and Stat5 [1]. These signaling cascades must be tightly regulated which mainly occurs through ubiquitin ligase-mediated receptor degradation and through dephosphorylation mediated by specific protein tyrosine phosphatases. Oncogenic mutations in cancer can lead to decreased receptor tyrosine kinases ubiquitination, either due to mutations of the binding sites for ubiquitin ligases in the receptors themselves, or through inactivating mutations in the ubiquitin ligases [10].

In this study, we examine the role of SLAP in Flt3 signaling pathways. We provide the evidence that SLAP directly associates with Flt3 in response to ligand stimulation and regulates receptor ubiquitination and degradation in a Cbl-dependent manner. Furthermore, selective knockdown of SLAP reduces FL-induced Akt, Erk and p38 activation.

## Materials and Methods

### Reagents and Antibodies

The transfection reagents Lipofectamine 2000 and jetPEI were from Invitrogen and Polyplus-transfection, respectively. Cycloheximide was from Sigma. The phospho-tyrosine antibody 4G10 was from Millipore and the ubiquitin antibody was from Covance Research Products. Flt3 antibody was described previously [11]. Shc, phospho-p38 and p38 antibodies were from BD Transduction Laboratories. Phospho-Akt antibody was from Epitomics. Polyclonal antibodies against SLAP, Gab2, Shp2, Akt, phospho-Erk and Erk were purchased from Santa Cruz Biotechnology. Phycoerythrin (PE)-labeled Flt3 antibody was from BD Biosciences. Horseradish peroxidase-coupled secondary anti-mouse and anti-rabbit antibodies were from Invitrogen.

### Expression Constructs

The pcDNA3-Flt3-WT, pcDNA3-Flt3-ITD and pMSCV-Flt3-WT constructs were previously described [11]. The pcDNA3-SLAP-FLAG was constructed by sub-cloning of the full-length open reading frame of SLAP into pcDNA3 vector. Flt3 mutant, Flt3-Y589F/Y591F/Y599F was generated by site-directed mutagenesis using QuikChange mutagenesis XL kit (Stratagene, La Jolla, CA).

### Cell Culture

Ba/F3 and COS-1 cell lines were purchased from DSMZ (www.dsmz.de). Ba/F3 cells were cultured in RPMI 1640 medium supplemented with 10% heat-inactivated fetal bovine serum (FBS), 100 units/ml penicillin, 100 µg/ml streptomycin and 10 ng/ml recombinant murine interleukin-3 (IL-3). COS-1 and EcoPack cells were grown in Dulbecco's modified Eagle's medium supplemented with 10% FBS, 100 units/ml penicillin and 100 µg/ml streptomycin.

### Transient Transfection

COS-1 cells were transiently transfected using JetPEI according to the manufacturer’s protocol. Cells were serum-starved overnight 24 hours after transfection and then stimulated at 37°C 100 ng/ml of FL (Prospec Tany) for the indicated time.

### Stable Transfection of Ba/F3 Cells

Packaging EcoPack cells were transfected with pMSCV-Flt3-WT construct using Lipofectamine 2000. Virus-containing supernatants were collected 72 hours after transfection. Ba/F3 cells were then infected with virus particles followed by a 2-week selection in 1.2 µg/ml puromycin. Expression of Flt3 was confirmed by flow cytometry. Flt3 transfected Ba/F3 cells were then further transfected with the SLAP shRNA and control shRNA constructs by electroporation. Cells were selected with 0.8 mg/ml G-418 for 2 weeks and SLAP knockdown was examined by Western blotting. Ba/F3 cells were serum-starved for 4 hours in RPMI-1640 medium without serum and cytokines, and then stimulated with 100 ng/ml of FL at 37°C for the indicated time.

### Affinity Fishing of SLAP with Immobilized Peptides

Phosphopeptides corresponding to the tyrosine motifs of Flt3 intracellular domain (or their non-phosphorylated counterpart; for sequence see [12], [13]) were immobilized on UltraLink beads(Thermo Scientific) according to the manufacturer’s instructions. Immobilized peptide slurry (50 µl) was incubated at 4°C for 2 hours with SLAP-transfected COS-1 cell lysates. Peptide bound proteins were then processed for Western blotting.

### Immunoprecipitation and Western Blotting

After stimulation, cells were washed once with ice-cold PBS and lysed in 1% Triton X100 lysis buffer. Lysates were then processed for immunoprecipitation and Western blotting as previously described [14]. Immunodetection was performed by enhanced chemoluminescence using Immobilon Western chemoluminescent horseradish peroxidase substrate (Millipore Corporation, Milford, MA, USA) and a CCD camera (LAS-3000, Fujifilm, Tokyo, Japan). The signal intensity was quantified by Multi-Gauge software (Fujifilm).

### Co-localization Study

Ba/F3 cells transfected with Flt3 and SLAP-FLAG were after starvation stimulated or not with FL for 5 minutes. Cells were then fixed, permeabilized and blocked followed by staining with fluorophore-conjugated anti-Flt3 and anti-FLAG. Nuclei were stained with DAPI. Localization of Flt3 and SLAP was visualized with Carl Zeiss LSM 710 Laser Scanning Microscope. Co-localization was measured using CoLocalizer Pro 2.7.1.

### Receptor Degradation

Cells were incubated with 100 µg/ml of cycloheximide for 4 hours at 37°C in RPMI-1640 medium without serum and cytokines. Cells were then incubated with or without 100 ng/ml of FL for 30 minutes followed by lysis and immunoprecipitation with an anti-Flt3 antibody. Samples were assessed by SDS-PAGE and Western blotting. Signal intensity was quantified by Multi-Gauge software (Fujifilm).

### Microarray Data Analysis

The microarray dataset for human patient samples from different cancers (GSE9476, GSE5550, GSE2550, GSE5788, GSE4290, GSE2223, GSE20437, GSE6691, GSE4115, GSE6919 and GSE8671) were retrieved from NCBI GEO site. Each data set contains relative expression of SLAP mRNA. The gene expression profiles of 18 acute promyelocytic leukemia (APL) patients (GSE2550) were used for analysis. Detailed information about patient samples was described elsewhere [15]. The data were then normalized and scaled using manual Excel equations.

### Statistical Analysis

Where appropriate, data were expressed as the mean ± SE. Data was analyzed by one-way ANOVA with Bonferroni’s or Dunnett’s post-tests using GraphPad Prism 5.0. Statistical significance was set at P<0.05.

## Results

### SLAP Interacts with Flt3 in a Phosphorylation Dependent Manner

**Figure 1:**
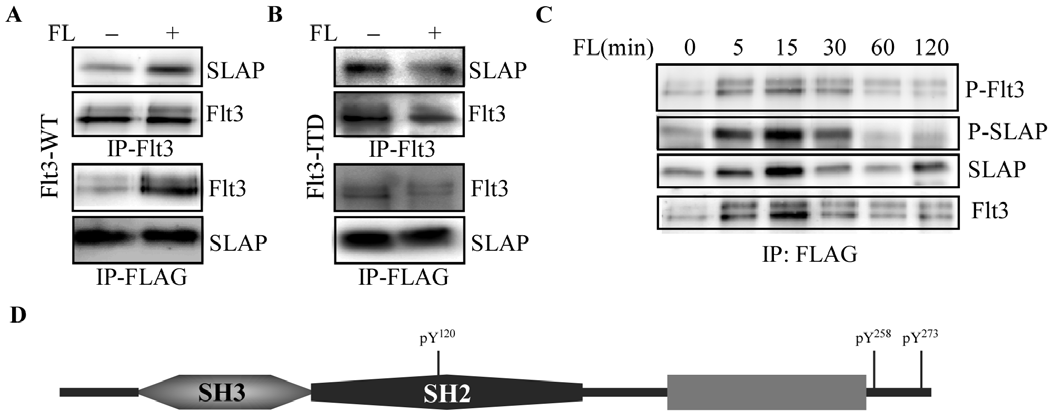
SLAP interacts with Flt3 in response to FL-stimulation. (A) COS-1 cells were co-transfected with pcDNA3-SLAP-FLAG and pcDNA3-Flt3-WT expression plasmids. Twenty four hours after transfection, cells were serum-starved overnight and stimulated by FL or not for 5 minutes before lysis. Cell lysates were immunoprecipitated with anti-Flt3 or anti-FLAG antibody followed by Western blotting analysis. (B) SLAP-FLAG and Flt3-ITD transfected COS-1 cells were stimulated for 5 minutes after overnight starvation. Cells were then processed for immunoprecipitation and Western blotting analysis. (C) SLAP-FLAG and Flt3-WT transfected COS-1 cells were stimulated for different time points after overnight starvation. Cells were then processed for immunoprecipitation with an anti-FLAG antibody and Western blotting analysis. Total Flt3 and SLAP phosphorylation was detected by 4G10 and the membrane was further probed with SLAP and Flt3 antibodies. (D) Structure of SLAP showing different domains and known tyrosine phosphorylation sites.

SLAP has been shown to associate with proximal components of the TCR and BCR signaling complexes [16]. This association is mediated through the SH2 domain of SLAP and tyrosine-phosphorylated residues of the receptors. To determine whether SLAP interacts with receptor tyrosine kinase Flt3, we transiently expressed SLAP and Flt3 in COS-1 cells. FL stimulation induced strong association of SLAP with Flt3 (Fig. 1A). Furthermore, SLAP was found to be constitutively associated with Flt3-ITD (Fig. 1B). However, we were unable to detect the endogenous Flt3-SLAP association probably due to the limited sensitivity of the antibodies used. A kinase dead mutant of Flt3 was unable to interact with SLAP (data not shown) and SLAP was found to be phosphorylated in response to FL (Fig. 1C). Similar to the kinetics of Flt3 activation, Flt3-SLAP interaction was rapid and reached a maximum after 5 min of ligand stimulation and was stable at least for 2 hours (Fig. 1C).

### SLAP Interacts with Multiple Flt3 Phosphotyrosine Residues Independent to Src Binding Sites

**Figure 2:**
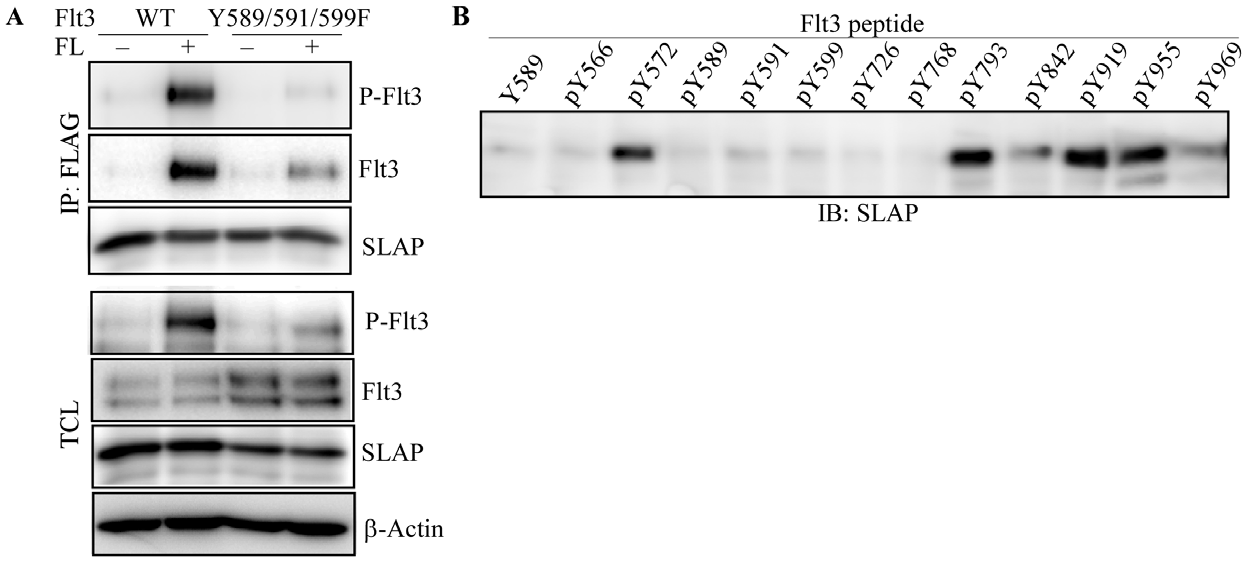
SLAP interacts with multiple Flt3 phosphotyrosine residues independent of the Src interaction sites. (A) COS-1 cells were co-transfected with pcDNA3-SLAP-FLAG and pcDNA3-Flt3-Y589/591/599F expression vectors. Twenty four hours after transfection, cells were serum-starved overnight and stimulated by FL or not for 5 minutes before lysis. Cell lysates were immunoprecipitated with anti-FLAG antibody followed by Western blotting analysis. (B) Phospho-peptides corresponding to 12 tyrosine phosphorylation sites in Flt3 were immobilized on UltraLink. Peptide-bound slurry was incubated with SLAP-transfected COS-1 cell lysates and pulled-down proteins were then processed for Western blotting using an anti-SLAP antibody.

As SLAP shows considerable similarity with Src, we first checked whether SLAP could bind through Src binding sites in Flt3. Three phospho-tyrosine residues 589, 591 and 599 are known as Src binding sites in Flt3 [17]. Thus we mutated those three tyrosine residues to phenylalanine. Co-transfection study showed that SLAP-Flt3 interaction is reduced in mutant Flt3 compared to wild-type Flt3 (Fig. 2A). However, Flt3 tyrosine phosphorylation was also reduced in mutant cells suggesting that reduction of SLAP-Flt3 interaction is not due to the loss of binding sites but to reduction of kinase activity. To resolve this problem we used the phospho-peptide fishing method. At least 12 tyrosine residues in Flt3 intracellular domain are predicted to be phosphorylated upon activation. The individual peptides were immobilized on UltraLink and subjected to pull down SLAP from cell lysates. Western blotting with an anti-SLAP antibody identified pY572, pY793, pY919 and pY955 residues as potential SLAP binding sites in Flt3 (Fig. 2B).

### SLAP is Expressed in Mouse pro-B Ba/F3 Cells

**Figure 3:**
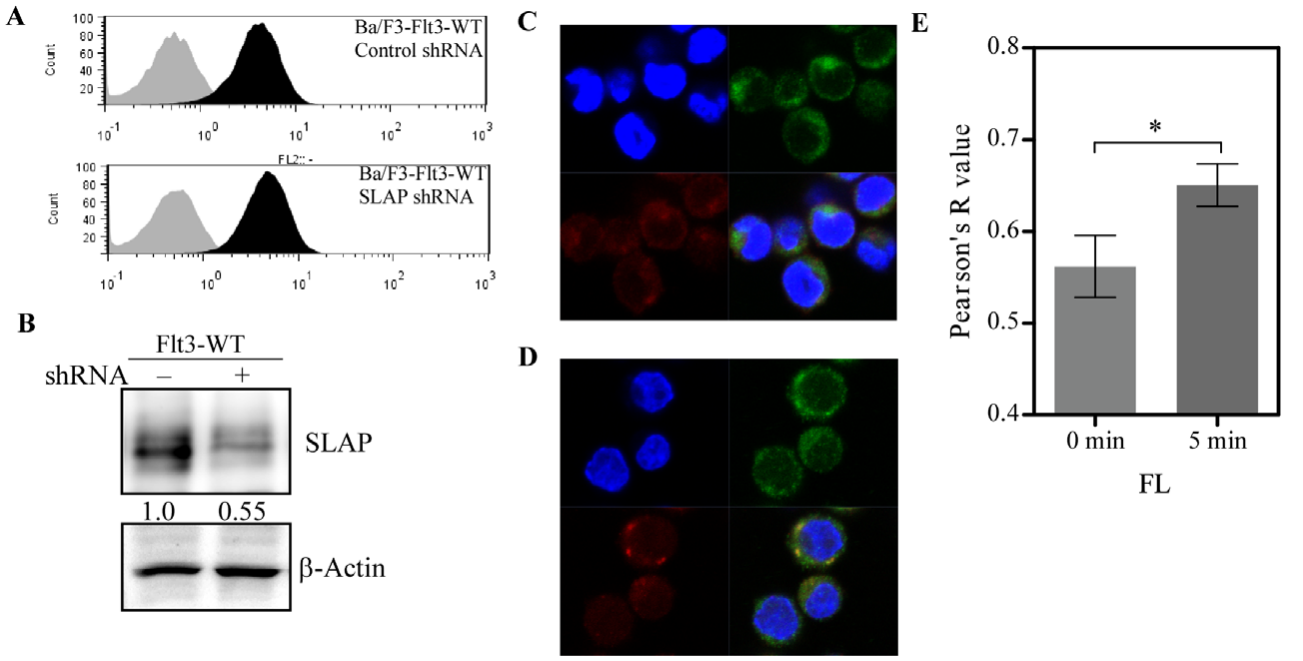
Expression of SLAP and Flt3 in Ba/F3 cells. (A) Stably transfected Ba/F3 cells were labeled with isotyp-matched control or PE-conjugated Flt3 antibody and analyzed by flow cytometry. Gray area indicates cells labeled with isotype-matched control and black area indicates cells labeled with Flt3 antibody. (B) Lysates of stably transfected Ba/F3-Flt3-WT cells with SLAP shRNA or control shRNA were immunoprecipitated with an anti-SLAP antibody and analyzed by Western blotting. (C) Ba/F3 cells transfected with Flt3-WT and SLAP-FLAG were stained with fluorophore-conjugated primary antibodies. (D) Ba/F3 cells transfected with Flt3-WT and SLAP-FLAG were stimulated with FL for 5 minutes and then stained with fluorophore-conjugated primary antibodies. Blue: DAPI, green: SLAP-FLAG and red: Flt3. (E) Co-localization of SLAP and Flt3 was determined using CoLocalizer Pro 2.7.1.

SLAP is expressed in a wide range of cell types including B-cells, T-cells, macrophages and mast cells [6], [18], [19]. We observed abundant SLAP expression in Ba/F3 and 32D cell lines (data not shown). In order to investigate the role of SLAP in Flt3 signaling we generated Ba/F3 cell lines stably transfected with Flt3-WT along with SLAP shRNA or control shRNA (Ba/F3 cells lack endogenous Flt3 expression). Flt3 expression levels were verified with flow cytometry (Fig. 3A) and knockdown of SLAP was checked by immunoblotting (Fig. 3B). We could achieve around 50% SLAP knockdown in our Ba/F3 system probably due to the moderate transfection efficiency. We also analyzed subcellular localization of SLAP. SLAP was predominantly found to be localized to the cell membrane (Fig. 3C) and FL-stimulation significantly increased co-localization with Flt3 (Fig. 3D and 3E).

### Phosphorylation of Akt, Erk and p38 was Reduced in SLAP Depleted Cells

**Figure 4:**
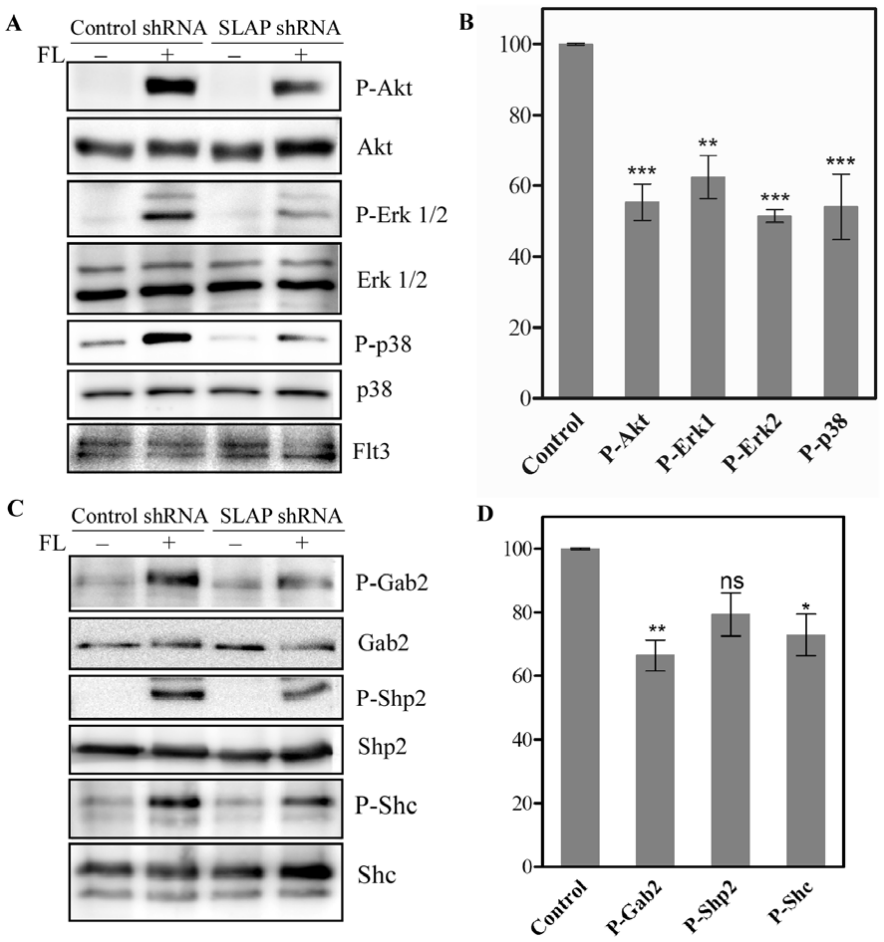
Effects of SLAP on FL-induced Akt, Erk and p38 activation. Ba/F3-Flt3 cells with SLAP shRNA or control shRNA were treated with 100 ng/ml FL or not for 5 minutes before lysis. (A) Total cell lysates were separated by SDS-PAGE and membranes were probed with phospho-specific antibodies. Membranes were then stripped and reprobed with respective antibodies to show the loading. (B) Signal intensities from three independent experiments of Fig. A were quantified using Multi-Gauge software.(C) Cell lysates were immunoprecipitated with respective antibodies and analyzed by Western blotting. (D) Signal intensities from three independent experiments of Fig. C were quantified using multi-gauge software.

Ligand binding to the Flt3 receptor initiates dimerization of receptor and activation of several signaling pathways including the PI3K-Akt pathway, the Ras/Erk pathway and the p38 pathway [1]. To study the effect of SLAP on these pathways we measured phosphorylation of Akt, Erk and p38 by Western blotting using phospho-specific antibodies. Selective knockdown of SLAP in Ba/F3-Flt3-WT cells reduced FL-induced activation of Akt, Erk 1/2 and p38 (Fig. 4A). The intensity of individual bands were quantified and normalized to loading controls. A comparison between control shRNA and SLAP shRNA transfected cells revealed that SLAP knockdown significantly reduced FL-induced phosphorylation of Akt, Erk 1/2 and p38 (Fig. 4B).Activation of PI3K-Akt pathway by Flt3 in response to FL is mainly mediated through the adaptor protein Gab2 or the protein tyrosine phosphatase Shp2, while the MAPK pathway is triggered by tyrosine phosphorylation of Shc [1]. A decrease of Gab2, Shp2 and Shc tyrosine phosphorylation was observed upon SLAP depletion (Fig. 4C). This tyrosine phosphorylation was significant for Gab2 and Shc but not for Shp2 (Fig. 4D).

### SLAP is Involved in Ubiquitination and Degradation of Flt3 Receptor

**Figure 5:**
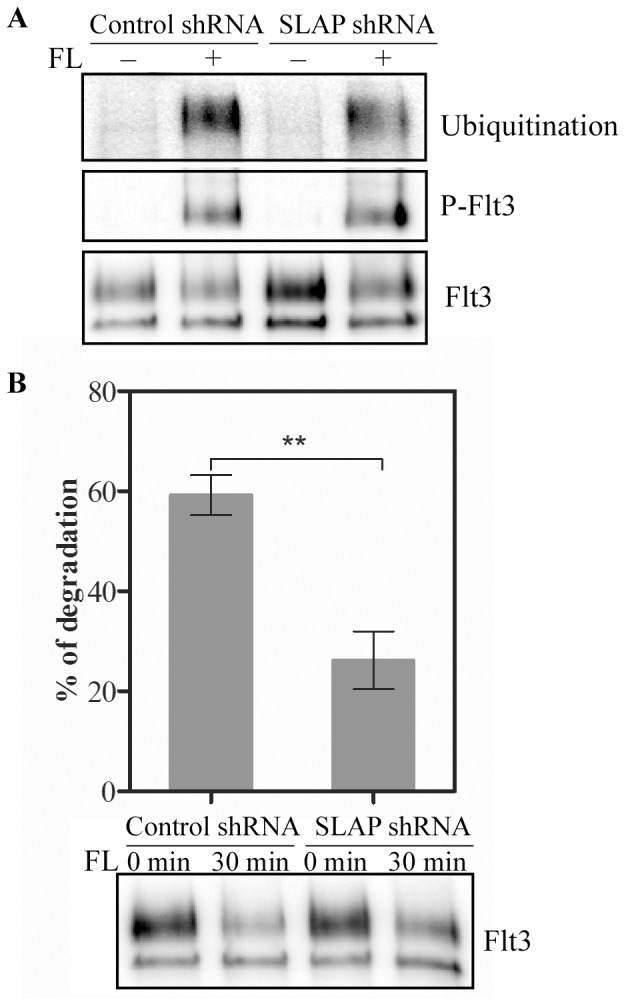
SLAP depletion decreases ubiquitination and degradation of Flt3 receptor. (A) Ba/F3-Flt3 cells with SLAP shRNA or control shRNA were serum-starved and stimulated with 100 ng/ml FL for 5 minutes. Cells were lysed and lysates were immunoprecipitated with an anti-Flt3 antibody followed by Western blotting analysis. (B) Ba/F3-Flt3 cells with SLAP shRNA or control shRNA were serum-starved and preincubated with cycloheximide for 4 hours. Cells were then stimulated with 100 ng/ml FL for 30 minutes before lysis and immunoprecipitated with an anti-Flt3 antibody. After Western blotting analysis signal intensities were quantified using Multi-Gauge software.

SLAP regulates the stability of receptors. In response to the external stimuli SLAP binds to TCR and BCR promoting ubiquitination and degradation of the receptors [6], [19]. In order to assess the impact of SLAP-Flt3 interaction on receptor stability we studied ubiquitination and degradation of Flt3 upon ligand stimulation. Loss of SLAP reduced ubiquitination of Flt3 in Ba/F3-Flt3-WT cells after 5 minutes of ligand stimulation (Fig. 5A). As receptor ubiquitination promotes degradation of the receptor, we stimulated cells with FL for 30 minutes and measured Flt3 degradation. Similar to the ubiquitination, Flt3 degradation was reduced in SLAP-depleted cells (Fig. 5B).

### SLAP Increases Flt3-mediated Cbl Tyrosine Phosphorylation

**Figure 6:**
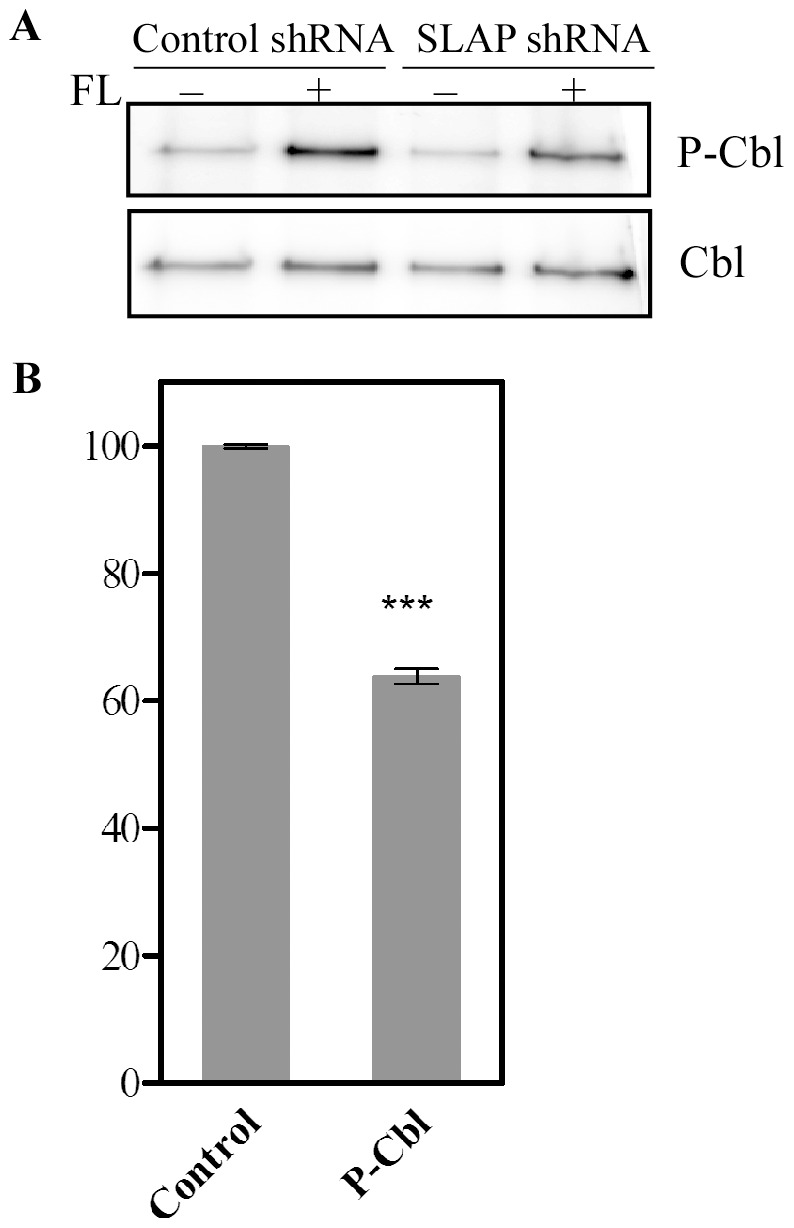
SLAP depletion decreases Cbl phosphorylation. Ba/F3-Flt3 cells with SLAP shRNA or control shRNA were treated with 100 ng/ml FL or not for 5 minutes before lysis. (A) Cell lysates were immunoprecipitated with an anti-Cbl antibody and analyzed by Western blotting. (B) Signal intensities from three independent experiments were quantified using multi-gauge software.

The E3 ubiquitin ligase Cbl associates with Flt3 which leads to ubiquitination and degradation of the receptor in proteasomes. SLAP has also been shown to regulate receptor ubiquitination in a Cbl-dependent manner [6], [16]. As we observed that SLAP depletion resulted in both reduction of ubiquitination and degradation of Flt3, we tested whether this effect was mediated by Cbl. We observed that SLAP knockdown caused a reduction of Cbl tyrosine phosphorylation which is required for Cbl activation (Fig. 6A). This reduction was highly significant (P<0.0001) suggesting that Cbl is required for SLAP mediated receptor ubiquitination (Fig. 6B).

### SLAP is Differentially Expresses in Different Cancers

**Figure 7:**
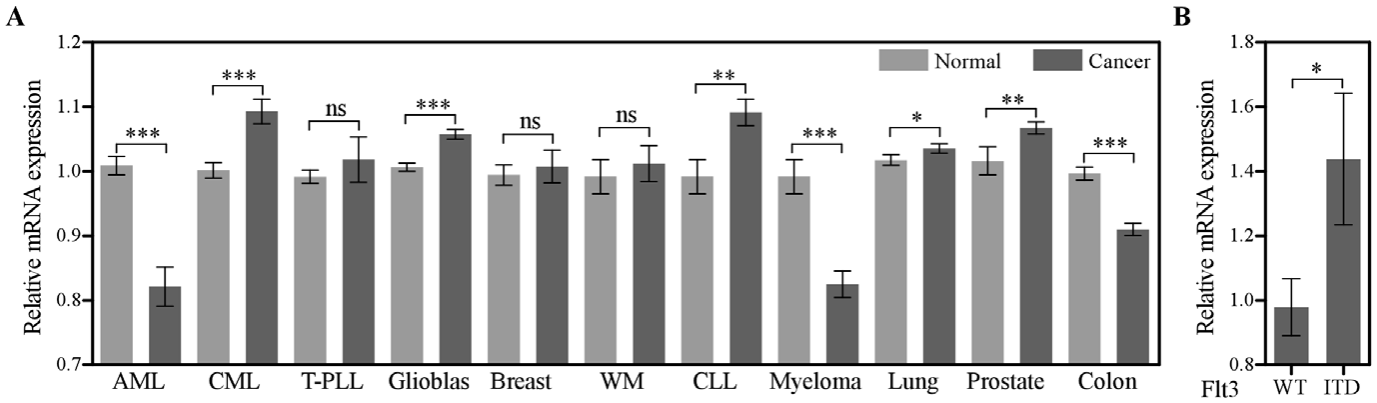
SLAP mRNA expression in leukemia patient samples. (A) SLAP mRNA expression was analyzed from microarray data of different patient samples and corresponding healthy donors. (B) SLAP mRNA expression was analyzed from the microarray dataset of acute promyelocytic leukemia (APL) patients carrying either wild-type FLT3 or FLT3-ITD mutation. Error bar shows SEM, and t-test was performed to determine significance. CML, Chronic myeloid leukemia; T-PLL, T-cell-prolymphocytic leukemia; WM, Waldenström's macroglobulinemia; CLL, Chronic lymphocytic leukemia.

According to the Human Protein Atlas database [20] SLAP is highly expressed in the gastrointestinal-tract and lung but moderately expressed in liver, pancreas, skin and other soft tissues. SLAP also displays differential expression patterns in cancer tissues. For example, SLAP expression is increased in pancreatic cancer while decreased in stomach cancer. This suggests that SLAP expression might be tissue specific. To test this idea we analyzed microarray data from different cancer patient samples and corresponding healthy donors. We observed that SLAP expression is significantly decreased in AML, myeloma and colon cancer, while it remains unchanged in T-PLL, breast cancer as well as WM (Fig. 7A). On the other hand, SLAP expression is significantly increased in CML, glioblastomas, CLL, lung cancer and prostate cancer (Fig. 7A). Although SLAP displays an opposite expression level in AML and CML, the expression was significantly increased in APL patients carrying an Flt3-ITD mutation in comparison to patients carrying wild-type Flt3 (Fig. 7B).

### The Global Protein Interaction Network of SLAP

**Figure 8:**
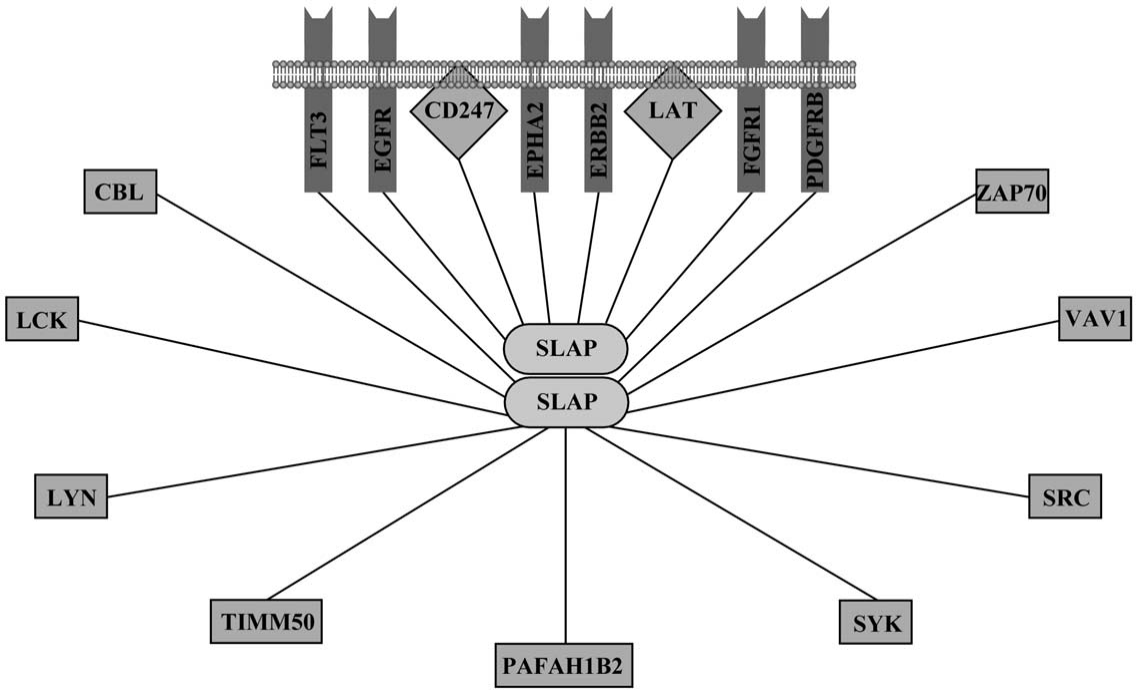
Global protein interaction network of SLAP. Global protein interaction network for SLAP was determined using PSICQUIC View, Gene Network Central and Cytoscape. Network was redrawn using Canvas. The Flt3-SLAP interaction is included from this study.

The global protein network of SLAP was analyzed by using PSICQUIC View, Gene Network Central and Cytoscape. SLAP interacts with multiple receptor and non-receptor protein tyrosine kinases as well as adaptor proteins (Fig. 8). Thus we suggest that SLAP might regulate multiple signaling pathways. A brief inspection of NCI/Nature Pathway Interaction Database using Cytoscape revealed that SLAP regulates 40 different signaling events (data not shown).

## Discussion

Deregulated activation of RTKs is a common event in human cancers. Inappropriate ligand stimulation, overexpression due to genetic amplification, and activating mutations in the coding region induce aberrant activation of RTKs. Flt3 is highly expressed in AML and aberrant activation of Flt3 by oncogenic mutations has important functions in AML pathogenesis. To understand the mechanism that control Flt3 signaling, we investigated the role of SLAP in Flt3 signaling. We observed that SLAP interacts with Flt3 and this interaction regulates Flt3-dependent signaling pathways and also promoted ubiquitination and degradation of the receptor. SLAP interacts with receptors in both phosphorylation and non-phosphorylation dependent manner. For instance, PDGFR-SLAP complex was dependent on PDGFR phosphorylation [2], while SLAP associated with both the phosphorylated and non-phosphorylated form of EpoR [21]. We found that the SLAP-Flt3 interaction was phosphorylation dependent and that SLAP was tyrosine phosphorylated upon receptor activation. Three tyrosine phosphorylation sites are known to be phosphorylated in SLAP (Fig. 1D). SLAP regulates receptor signaling by linking receptors to Cbl, leading to ubiquitination and degradation of receptor [6], [16], [18], [22]. Cbl has been identified as a negative regulator of Flt3 signaling. In response to FL stimulation Cbl physically interacts with Flt3 and becomes tyrosine phosphorylated [5], [23]. Selective knockdown of SLAP in Ba/F3 cells displayed a significant reduction in Cbl phosphorylation as well as reduction in ubiquitination and degradation. This observation suggests that SLAP may cooperate with Cbl to promote ubiquitination of Flt3. Known binding sites for Cbl interaction in Flt3 are Y589 and Y599, which are also known as Src interaction sites, do not overlap with the SLAP binding sites in Flt3 [5], [17]. Thus, SLAP does not compete with either Cbl or Src for association to Flt3 but rather enhances the Cbl-Flt3 interaction.

SLAP creates dimers through its carboxy-terminal tails and this dimerization is required for NAFT-AP1 promoter activation [24]. Enforced expression of SLAP inhibited cytokine receptor EpoR mediated STAT5 activation, while it failed to inhibit Epo-induced Erk 1/2 activation [21] and did not diminish the extent of phosphorylation of SFKs such as Lyn and Src [25]. SLAP is also known to associate with cytosolic tyrosine kinases such as Syk and ZAP70 through its SH2 domain [24]. These studies suggest that SLAP regulates multiple signaling pathways by creating dimers and by linking receptors to cytosolic proteins. The presence of multiple domains in SLAP mediates these interactions. Selective knockdown of SLAP expression by shRNA affected FL-induced Akt, Erk 1/2 and p38 phosphorylation suggesting that SLAP not only plays a role in regulation of receptor stability but also in Flt3 downstream signaling apart from receptor stability. Although FL-induced PI3K-Akt pathway activation is regulated by both SHP2 and Gab2 [1], SLAP modulates thePI3K-Akt pathway through Gab2. Tyrosine phosphorylation of Gab2 by either SFKs [1] and Syk [26] is needed for its activation. Reduction of Shc-tyrosine phosphorylation in SLAP depleted cells further demonstrates that SFKs are involved in SLAP-mediated signal transduction. Thus SLAP potentiates Flt3 downstream signaling despite the fact thatit reduces receptor stability.

The SLAP shows differential expression in different tissues suggesting that SLAP is involved in tissue specific cellular functions. Although overall SLAP expression is decreased in AML patients, a stage specific expression was observed in patients. In the M3 class AML, 37% of APL patients carry an oncogenic Flt3 mutation [27]. The SLAP expression was significantly elevated in patients carrying the Flt3-ITD mutation as compared to the Flt3-WT, suggesting that SLAP plays a role in Flt3-ITD signaling. The expression data generated from microarray analysis always suffer from limitations in reliability [28], thus it is of interest to validate such observations in APL patients on both the mRNA and protein levels using qPCR and Western blotting.

### Conclusions

**Figure 9:**
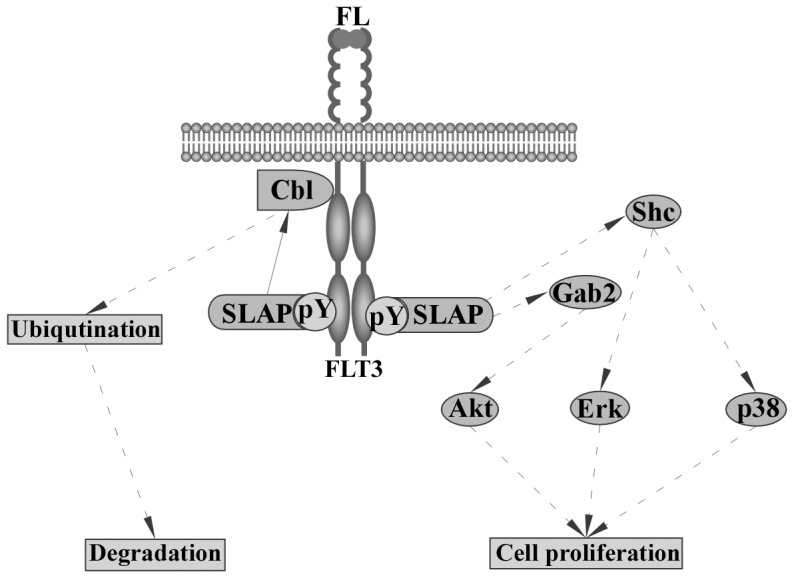
Schematic representation of SLAP signaling in response to FL stimulation. SLAP interacts with Flt3 in response to FL stimulation and recruits Cbl leading to receptor degradation. Interaction of SLAP with Flt3 also leads to activation of Akt, Erk and p38 followed by cell proliferation.

The effects of SLAP on receptor tyrosine kinase signaling are more complex than previously recognized. Our data suggest that the stability of Flt3 is partially controlled by SLAP in a Cbl-dependent manner and that SLAP regulates Flt3 downstream signaling through SFKs (Fig. 9). SLAP displays tissue specific expression. While different levels of expression was observed in different cancers, an upregulation of SLAP expression was found in patients carrying the Flt3-ITD mutation as compared to wild-type Flt3. Thus, we suggest that SLAP plays a key role in Flt3 signaling.
